# Experiences of Newly Graduate Nurses With Workplace Violence: A Qualitative Meta‐Synthesis

**DOI:** 10.1155/jonm/4496213

**Published:** 2026-03-08

**Authors:** Gilny Rantung, Yaping Zhong

**Affiliations:** ^1^ Faculty of Nursing, Indonesian Adventist University, Kabupaten Bandung Barat, Jawa Barat, Indonesia; ^2^ Monash Nursing and Midwifery, Monash University, Clayton Victoria, Australia, monash.ac.za

**Keywords:** meta synthesis, new graduate nurses, workplace violence

## Abstract

**Aims:**

To explore and integrate qualitative evidence on the experiences of workplace violence as reported in studies involving newly graduated nurses, with a focus on its forms, coping mechanisms and resulting effects.

**Design:**

Qualitative meta‐synthesis.

**Methods:**

The review included qualitative studies relevant to the topic. A systematic search was conducted from database inception to September 2024. The quality of the included studies was appraised using the Joanna Briggs Institute Critical Appraisal Checklist for Qualitative Research. Two reviewers independently screened titles, abstracts, and full texts before inclusion. Data analysis was conducted using a thematic synthesis approach.

**Data Sources:**

A systematic literature search was conducted in eight databases: CINAHL, Scopus, Emcare, Web of Science, ProQuest, Embase, MEDLINE, and PsycINFO.

**Results:**

Eighteen studies were included. The synthesis identified five forms of workplace violence, four coping strategies, and five consequence domains. These findings informed a conceptual model illustrating workplace violence as a cyclical, systemically reinforced process linking its manifestations, the coping responses employed by new nurses and the resulting personal and professional impacts.

**Conclusion:**

New nurses experienced cyclical and systemic workplace violence. Their coping mechanisms provided temporary relief but did not address the root causes of workplace hostility. This led to lasting negative effects and reinforced further workplace violence, requiring both individual and organisational interventions to disrupt the cycle.

**Summary**
• Implications for the Profession
◦ To improve new nurse retention, job satisfaction and patient care quality, healthcare organisations should move beyond individual coping strategies and focus on systemic interventions. By fostering a culture of respect, implementing robust antibullying policies and integrating workplace violence (WPV) prevention into education, organisations can enhance staff well‐being and patient care outcomes.
• Impact
◦ The meta‐synthesis highlights that WPV against newly graduated nurses is systemically embedded within healthcare settings and sustained by hierarchical structures and a culture of silence, which shapes how new nurses experience and respond to aggression in the workplace. This normalisation of WPV in some institutions, rather than it being viewed as isolated incidents, highlights the need for change.
• Reporting Method
◦ The authors adhered to the ENTREQ guidelines.
• No patient or public contribution.
• What does this paper contribute to the wider global clinical community?
◦ This study highlights the systemic nature of WPV, showing it is embedded and sustained by structures and culture, requiring systemic changes.
◦ This study provides a cyclical model of WPV, linking inequities and aggression to coping mechanisms and negative outcomes, which shows the need for multilevel interventions.
◦ Systemic interventions are emphasised to improve nurse well‐being.

## 1. Introduction

Nursing is a demanding profession that requires technical expertise, emotional resilience, and strong interpersonal skills to deliver high‐quality patient care [1–3]. Nurses operate in complex and high‐pressure healthcare environments, providing critical care while navigating the demands of patient advocacy, interdisciplinary collaboration and ethical decision‐making [4]. Their role extends beyond clinical responsibilities to include health education, emotional support and crisis management, making nursing both a rewarding and challenging career [5]. Alongside workload and emotional strain, workplace violence (WPV) has emerged as a major occupational hazard for nurses, contributing substantially to work‐related stress and burnout [6, 7]. WPV, including physical assault, verbal abuse, bullying, and harassment, is associated with anxiety, depression, moral distress, diminished job satisfaction and increased turnover intentions and may compromise patient safety and the stability of the nursing workforce [8–10].

Newly graduated nurses play a vital role in healthcare systems, yet their transition from student to professional practice is fraught with challenges [11]. This period is characterised by a steep learning curve, role ambiguity, and increased workload, which can impact their confidence, professional identity and well‐being [12]. During this vulnerable transition, WPV is particularly damaging: New nurses often occupy lower hierarchical positions, have limited clinical experience, and may feel unable to speak up or challenge unsafe or hostile behaviours [13, 14]. Exposure to bullying, horizontal violence, and incivility in the early stages of practice can erode professional confidence, disrupt the development of a positive nursing identity and contribute to early career attrition [15, 16]. These impacts extend beyond individual nurses, affecting team functioning, quality of care and long‐term retention within the profession, underscoring the need to understand better how newly graduated nurses experience and respond to WPV.

## 2. The Review

WPV in nursing is broadly defined as any act or threat of verbal or physical violence, harassment, intimidation or disruptive behaviour that occurs in the workplace [6]. It encompasses a range of harmful behaviours, including verbal aggression, bullying, incivility, physical violence and sexual harassment [17]. According to the *Framework Guidelines for Addressing WPV in the Health Sector*, a joint program by the authors in [18], WPV refers to any incident in which healthcare workers are abused, threatened or assaulted in work‐related circumstances, including commuting to and from work, posing risks to their safety, well‐being and health. WPV includes physical violence, such as hitting, kicking and sexual assault, as well as psychological violence, such as bullying, harassment and intimidation, which can severely impact professional dignity and mental health [18].

WPV can be perpetrated by patients, visitors, colleagues or supervisors, creating a hostile work environment that negatively impacts nurses’ professional and personal well‐being [16]. A global meta‐analysis underscores the severity of WPV, reporting that approximately 62% of healthcare workers experienced violence from patients or visitors in the past year [19]. In a cross‐sectional study of nurses across five European countries, over 54% reported experiencing nonphysical violence, while 20% had been subjected to physical assaults [8]. Alarmingly, 70% of these incidents went uninvestigated, reflecting both the magnitude of WPV and the systemic underreporting that perpetuates the issue [8]. Research suggests that negative workplace behaviours—exclusion, intimidation and excessive scrutiny—can erode job satisfaction, heighten emotional distress and contribute to burnout [20]. Moreover, the repercussions of WPV extend beyond individual nurses, jeopardising patient care quality and workforce sustainability [9, 21].

While previous research has extensively examined WPV in healthcare settings, much of the literature relies on quantitative studies that primarily focus on prevalence rates and statistical trends [10, 14]. However, qualitative research offers an essential perspective by uncovering the lived experiences of nurses who encounter WPV, providing insight into the psychosocial and professional consequences that cannot be captured through numerical data alone. Narratives from qualitative studies reveal how new graduated nurses interpret, navigate and internalise WPV, shedding light on the emotional toll, coping mechanisms and workplace cultures that either enable or mitigate violence [15, 22, 23].

Despite the growing awareness of WPV, there remains a lack of comprehensive synthesis of qualitative evidence on how new nurses experience and respond to WPV. Most existing research examines WPV through individual studies with varying definitions, contextual factors and theoretical underpinnings, making it difficult to develop a holistic understanding of the phenomenon. By conducting a meta‐synthesis of qualitative studies, this research aims to provide a deeper insight into the lived experiences of new graduated nurses facing WPV, focussing on its manifestations, coping strategies and resultant impacts. Understanding these narratives contributes to a broader discussion on the cultural, organisational and interpersonal dimensions of WPV in nursing, offering a richer and more nuanced exploration of how WPV affects new nurses across different healthcare settings.

## 3. Aim

To explore and synthesise qualitative evidence on the experiences of WPV as reported in studies of newly graduated nurses, focussing on its manifestations, coping strategies and resultant impacts.

## 4. Methods/Methodology

### 4.1. Design

This study employed a qualitative meta‐synthesis (QMS) approach, which integrates the findings from primary qualitative studies to generate a deeper understanding of a phenomenon. By identifying patterns and relationships that transcend individual studies, QMS enables researchers to derive new insights and a broader perspective on the complex dynamics of WPV [24, 25].

This meta‐synthesis followed four structured stages: (a) conducting a systematic and comprehensive search to identify and extract relevant articles for analysis, (b) appraising the included studies to gain a comprehensive understanding of their findings and evaluate their scientific credibility and relevance, (c) classifying the extracted findings based on their level of interpretation to ensure a nuanced analysis, and (d) synthesising the findings to integrate and interpret them holistically [26]. The protocol of the review is registered in the International Prospective Register of Systematic Reviews (PROSPERO), and the registration number is CRD42024586849. A systematic review of the qualitative study was reported in accordance with the ENTREQ Checklist’s guidelines (Supporting File 1) [27].

### 4.2. Search Methods

A systematic search across eight electronic databases was conducted: CINAHL, Scopus, Emcare, Web of Science, ProQuest, Embase, MEDLINE and PsycINFO. The search strategy included three main concepts: “new nurse”, “WPV” and “qualitative”. The search strategy was developed iteratively by the review team, drawing on prior reviews of WPV in healthcare and database‐specific subject headings. For each database, synonyms and related terms for the three core concepts were identified, combined with Boolean operators, and piloted in CINAHL and MEDLINE before being adapted to the remaining databases. No date limits were applied. To enhance comprehensiveness, we also screened the reference lists of all included studies and relevant reviews. For each database, both free‐text searches and searches using subject terms or Medical Subject Headings (MeSH), where applicable, were conducted independently. The results of these searches were then combined. An example of the specific search strategy is shown in Supporting File 2.

### 4.3. Inclusion and/or Exclusion Criteria

This review focused on qualitative studies that explored the experiences, perceptions or incidents of WPV or aggression among newly graduated nurses or those with up to 1 year of professional experience in healthcare settings. Eligible studies included any type of qualitative studies, including but not limited to phenomenology, grounded theory, ethnography or descriptive qualitative research. Only peer‐reviewed articles published in English were considered, and the review included studies published up to September 2024. Qualitative components of mixed‐methods studies were eligible when the qualitative data and analysis were reported separately and could be clearly extracted. Mixed‐methods studies that did not present independently analysable qualitative findings were excluded.

Studies were excluded if they focused on populations other than newly graduated nurses, such as nursing students, patients, families or nurses with more than 1 year of professional experience. Quantitative studies, mixed‐methods studies without separate qualitative data analysis, editorials, reviews, commentaries, opinion pieces, non–peer‐reviewed articles, conference abstracts or grey literature were also excluded. Additionally, studies addressing violence unrelated to the workplace or aggression in nonhealthcare settings were not included.

### 4.4. Search Outcome

A total of 1234 records were identified through database searches, including CINAHL (*n* = 289), Scopus (*n* = 275), Emcare (*n* = 195), Web of Science (*n* = 170), ProQuest (*n* = 134), Embase (*n* = 74), MEDLINE (*n* = 60) and PsycINFO (*n* = 37). After removing 613 duplicates, with 26 identified manually and 587 detected by Covidence systematic review software, 621 records remained for screening. Two reviewers (GR and YZ) independently screened the titles and abstracts of these records in Covidence (Covidence systematic review software, Veritas Health Innovation, Melbourne, Australia), applying the inclusion and exclusion criteria. Discrepancies were resolved through discussion among the researchers. As a result, 573 records were excluded.

**FIGURE 1:**
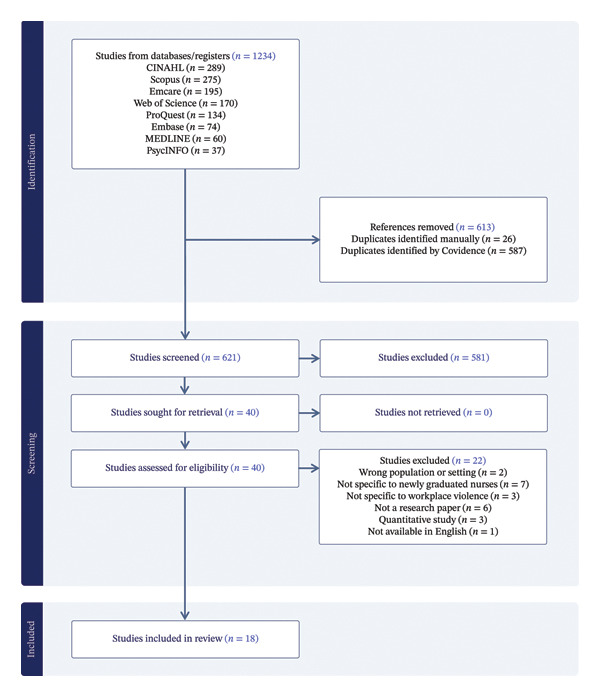
PRISMA flow diagram.

The remaining 40 articles were retrieved for full‐text review, all of which were successfully accessed. After full‐text assessment, 22 articles were excluded for reasons such as being literature reviews, quantitative studies, nonresearch papers, studies not conducted in healthcare settings, studies not available in English, studies not specific to newly graduated nurses or studies not focused on WPV. Ultimately, 18 studies met the inclusion criteria and were included in the review. A detailed flow of the search and selection process is presented in PRISMA flow diagram (Figure 1) [28].

### 4.5. Quality Appraisal

Studies were independently critically appraised for methodological quality, by two reviewers (GR and YZ) using the Joanna Briggs Institute Critical Appraisal tools for Systematic Reviews, Checklist for Qualitative Research [29], as reported in Table 1. Where Item 1 was recorded as N/A due to nonreporting of a philosophical perspective, rigour was appraised using congruity across Items 2–5. No studies were excluded after critical appraisal.

**TABLE 1:** Critical appraisal.

Author (year)	1	2	3	4	5	6	7	8	9	10	Overall appraisal
Halpin Y.; Terry L.M.; Curzio J. (2017)	N/A	Y	Y	Y	Y	N	N	Y	Y	Y	Include
Simons S.R.; Mawn B. (2010)	N/A	Y	Y	Y	Y	N	N	Y	Y	Y	Include
Madolo, Agrinette N.; Hloba, S.P. (2023)	N/A	Y	Y	Y	Y	U	N	Y	Y	Y	Include
Gillespie G.L.; et al. (2024)	N/A	Y	Y	Y	Y	N	N	Y	U	Y	Include
Baloyi, T.S.; et al. (2024)	N/A	Y	Y	N	Y	N	N	Y	Y	Y	Include
Alsalamah, Y.; Fawaz, M. (2023)	N	Y	Y	U	Y	U	N	Y	Y	Y	Include
Chachula, K.M.; et al. (2015)	N/A	Y	Y	Y	Y	N	N	Y	Y	Y	Include
Hover, L.A.; Williams, G.B. (2022)	N/A	Y	Y	Y	Y	N	N	Y	Y	Y	Include
Rosi, I.M.; et al. (2020)	N/A	Y	Y	Y	Y	N	N	Y	Y	Y	Include
Mammen, B.; Hills, D.J.; Lam, L. (2018)	N/A	Y	Y	Y	Y	N	N	Y	Y	Y	Include
Bry, A.; Wigert, H. (2022)	N/A	Y	Y	Y	Y	Y	U	Y	Y	Y	Include
Kerber, C.; et al. (2015)	N/A	Y	Y	U	Y	N	N	Y	Y	Y	Include
Krut, B.A.; et al. (2021)	N/A	Y	Y	Y	Y	N	N	Y	Y	Y	Include
Leong, Y.M.J.; Crossman, J. (2016)	Y	Y	Y	Y	Y	N	N	Y	Y	Y	Include
McKenzie, R.; et al. (2021)	N/A	Y	Y	Y	Y	N	N	Y	Y	Y	Include
Abrar, M.; et al. (2019)	N/A	Y	Y	Y	Y	N	N	Y	U	Y	Include
Mao, A.; et al. (2021)	Y	U	Y	N	Y	Y	U	Y	Y	Y	Include
Anusiewicz, C.V.; et al. (2020)	N/A	Y	Y	Y	Y	Y	U	Y	Y	Y	Include

*Note:* N/A = not reported/not assessable from the published report. Description of Questions: (1) Is there congruity between the stated philosophical perspective and the research methodology? (2) Is there congruity between the research methodology and the research question or objectives? (3) Is there congruity between the research methodology and the methods used to collect data? (4) Is there congruity between the research methodology and the representation and analysis of data? (5) Is there congruity between the research methodology and the interpretation of results? (6) Is there a statement locating the researcher culturally or theoretically? (7) Is the influence of the researcher on the research, and vice versa, addressed? (8) Are participants, and their voices, adequately represented? (9) Is the research ethical according to current criteria or, for recent studies, and is there evidence of ethical approval by an appropriate body? (10) Do the conclusions drawn in the research report flow from the analysis or interpretation of the data?Item 1 note: N/A indicates that the philosophical perspective was not reported; therefore, appraisal emphasised congruity across Items 2–5.

Abbreviations: Y = yes; N = no; U = unclear.

### 4.6. Data Analysis and Synthesis

A standardised data extraction form was developed by the review team, guided by the review aim and ENTREQ recommendations. The form was designed to capture information needed to compare study contexts and to address the review focus on forms of WPV, coping strategies and consequences. The data extraction process was conducted by GR and YZ, who systematically collected key information such as study purpose, participant characteristics, data collection methods, methodologies and main findings. This information was summarised in a structured table (Table 2). The thematic synthesis method by Thomas and Harden [30] guided the analysis, comprising line‐by‐line coding, grouping into descriptive themes and generating analytical themes to address the research objectives.

**TABLE 2:** Characteristics of the studies.

Author (year)	Country	Aims of study	Methodology	Data collection method	Setting	Participants
Halpin Y.; Terry L.M.; Curzio J. (2017)	UK	Investigate workplace stressors and stress experiences of newly qualified nurses during transition.	Mixed methods	Survey, semistructured interviews	Hospitals	Newly qualified nurses (*n* = 288, reduced to *n* = 14 for interviews)
Simons S.R.; Mawn B. (2010)	USA	Explore the experiences and perceptions of bullying among newly licenced registered nurses.	Qualitative descriptive	Open‐ended survey responses	Various healthcare settings	Newly licenced registered nurses (*n* = 184)
Madolo, Agrinette N.; Hloba, Siyathemba P. (2023)	South Africa	Explore the impact of bullying, staff shortages, and resource constraints on newly qualified nurses.	Qualitative descriptive	Semistructured interviews	Public hospitals	Newly qualified nurses (*n* = 19)
Gillespie G.L.; Tamsukhin S.M.; Galloway E.; Garde D.; Grubb P.L. (2024)	USA	Explore how newly licenced nurses manage workplace bullying behaviours.	Phenomenology	Semi‐structured interviews	Various healthcare settings	Newly licenced nurses (*n* = 24)
Baloyi, Tinyiko S.; Ramathuba, Dorah U.; Netshisaulu, Khathutshelo G. (2024)	South Africa	Explore the experiences of newly qualified registered nurses (R.683) regarding their workplace environment.	Qualitative descriptive	Focus group interviews	Public hospitals	Newly qualified registered nurses (*n* = 51)
Alsalamah, Y.; Fawaz, M. (2023)	Saudi Arabia	Explore facilitators and barriers for successful transition among new Saudi graduate nurses.	Phenomenology	Focus group interviews	University hospital	Newly graduated nurses (*n* = 35)
Chachula, K.M.; Myrick, F.; Yonge, O. (2015)	Canada	Explore the factors influencing newly graduated registered nurses’ decision to exit the nursing profession.	Grounded theory	Unstructured and semistructured interviews	Hospitals in Western Canada	Newly graduated nurses (*n* = 8)
Hover, L.A.; Williams, G.B. (2022)	USA	Explore the experiences of newly graduated nurses with lateral violence and their decision to remain in nursing.	Phenomenology	Semistructured interviews	Various healthcare settings in Arizona	Newly graduated nurses (*n* = 9)
Rosi, I.M.; Contiguglia, A.; Millama, K.R.; Rancati, S. (2020)	Italy	Explore the experiences of newly graduated nurses with horizontal violence in various healthcare settings.	Phenomenology	Face‐to‐face interviews	Hospitals and nursing homes in Milan, Italy	Newly graduated nurses (*n* = 21)
Mammen, B.; Hills, D.J.; Lam, L. (2018)	Australia	Explore newly qualified graduate nurses’ experiences of workplace incivility during their graduate nurse program.	Descriptive qualitative	Face‐to‐face in‐depth interviews	Australian hospitals	Newly graduated nurses (*n* = 8)
Bry, A.; Wigert, H. (2022)	Sweden	Describe the organisational climate and interpersonal interactions among NICU nurses.	Qualitative descriptive	Semistructured interviews	Level III Neonatal Intensive Care Unit (NICU), Sweden	Registered nurses (*n* = 13)
Kerber, C.; Woith, W.M.; Jenkins, S.H.; Astroth, K.S. (2015)	USA	To obtain a rich description of new nurses’ perceptions of incivility in the workplace to explore the impact of incivility on new nurses and patients.	Qualitative exploratory	Online questionnaire (open‐ended)	Various healthcare settings in the USA	Newly graduated nurses (*n* = 17)
Krut, B.A.; Laing, C.M.; Moules, N.J.; Estefan, A. (2021)	Canada	Explore the individual experiences of graduate nurses with horizontal violence (HV) in their first year of practice.	Thematic analysis	Semistructured interviews	Large urban hospital in Western Canada	Graduate nurses (*n* = 8). Four participants were GNs with 12 months of experience or less and four were RNs with varying years of experience that had been exposed to HV in their first 12 months of practice.
Leong, Y.M.J.; Crossman, J. (2016)	Singapore	Investigate the experiences of newly qualified nurses during their transition into professional roles, focussing on the concept of “tough love” by preceptors.	Constructivist grounded theory	Semistructured interviews, reflective journals	Five hospitals in Singapore	Newly qualified nurses (*n* = 26), preceptors (*n* = 5)
McKenzie, R.; Miller, S.; Cope, V.; Brand, G. (2021)	Australia	Explore the clinical and professional learning experiences of newly qualified registered graduate nurses in a Neonatal Intensive Care Unit (NICU) during their first 6 months of employment.	Narrative inquiry	Semistructured interviews	Two NICUs in Western Australia	Newly graduated registered nurses (*n* = 8)
Abrar, M.; Bashir, M.; Nureen, N.; Shahzadi, Q. (2019)	Pakistan	Explore the perceptions of newly graduated nurses regarding workplace incivility and its consequences on their professional and personal life.	Qualitative descriptive	In‐depth qualitative interviews	Public sector hospitals in Faisalabad, Pakistan	Newly graduated nurses (*n* = 10)
Mao, A.; Tam, H.L.; Cheong, P.L.; Van, I.K. (2021)	Macau	Explore the coping strategies of young nurses with lateral violence (LV) in clinical settings, framed from a feminist perspective.	Qualitative descriptive	Semistructured interviews	Hospitals in Macau, China	Nursing students and newly graduated nurses (*n* = 20)
Anusiewicz, C.V.; Ivankova, N.V.; Swiger, P.A.; Gillespie, G.L.; Li, P.; Patrician, P.A. (2020)	USA	Explore how workplace bullying influences nurses’ abilities to provide patient care.	Qualitative descriptive	Semistructured interviews	One hospital in southern USA	15 inpatient staff nurses (including new nurses)

GR conducted the initial line‐by‐line coding, treating author interpretations and participant quotes as a unified text to preserve original meaning and nuances. For example, codes such as “Isolation and neglect”, “Left alone to struggle” and “Emotional neglect” were grouped into descriptive themes such as “Isolation and neglect”. YZ reviewed the coding for consistency and contributed additional insights during the descriptive coding stage. The research team engaged in iterative discussions to refine the descriptive themes into broader analytical themes. For instance, “Public humiliation”, “Sarcastic comments and name‐calling” and “Physician‐Directed Verbal Hostility” were synthesised into the analytical theme “Verbal Aggression and Public Humiliation”. Discrepancies in interpretations were resolved collaboratively to ensure rigour. Meta‐themes were developed by synthesising analytical themes to capture common patterns and relationships across studies. These meta‐themes, reflecting forms, coping mechanisms and impacts of WPV, as reported in the studies on newly graduated nurses, are presented in the results (Table 3).

**TABLE 3:** Theme synthesis.

Themes and subtheme	Halpin et al. [31]	Simons and Mawn [32]	Madolo and Hloba [33]	Gillespie et al. [34]	Baloyi et al. [35]	Alsalamah and Fawaz [36]	Chachula et al. [37]	Hover and Williams [15]	Rosi et al. [38]	Mammen et al. [39]	Bry and Wigert [40]	Kerber et al. [41]	Krut et al. [42]	Leong and Crossman [43]	McKenzie et al. [23]	Abrar et al. [44]	Mao et al. [22]	Anusiewicz et al. [45]
Forms and Manifestations of Workplace Violence Experienced by New Nurses																		
• Systemic Hierarchical Hostility and Intimidation	✓	✓	✓	✓	✓		✓		✓		✓		✓	✓	✓	✓		✓
• Social Exclusion and Isolation	✓	✓	✓		✓				✓	✓	✓	✓	✓	✓				✓
• Verbal Aggression and Public Humiliation	✓	✓		✓	✓					✓		✓	✓	✓				
• Task Overload and Unfair Workload		✓	✓		✓					✓								
• Sexual Harassment and Physical Intimidation												✓				✓		
Coping Strategies of New Nurses Facing Workplace Violence																		
• Social and Professional Support	✓						✓	✓		✓			✓	✓			✓	
• Emotion‐Focused Coping								✓		✓				✓			✓	
• Avoidance Coping and Self‐Preservation				✓					✓	✓	✓	✓		✓		✓		✓
• Problem‐Focused Coping and Self‐Improvement							✓		✓	✓				✓			✓	
Consequences of Workplace Violence on Nurses’ Personal and Professional Lives																		
• Emotional and Psychological Impact	✓					✓	✓	✓		✓	✓			✓	✓			✓
• Erosion of Confidence and Professional Identity			✓	✓		✓	✓			✓			✓					✓
• Compromised Patient Care and Professional Standards	✓				✓				✓	✓	✓	✓						✓
• Career Regret and Challenges to Retention		✓	✓						✓	✓	✓		✓					
• Physical Health and Personal Life Disruptions									✓	✓			✓		✓			

## 5. Findings

This meta‐synthesis identified three key themes related to WPV experienced by new nurses: Forms and Manifestations of WPV, Coping Strategies and Consequences on Personal and Professional Lives. These themes directly addressed the review aim by mapping: (1) how WPV is experienced (Theme 1), (2) how new nurses respond to these experiences (Theme 2) and (3) what impacts follow (Theme 3). The themes are interrelated and sequential: forms of WPV commonly triggered coping responses, which in turn shaped subsequent consequences. Across studies, this pattern suggests a reinforcing cycle whereby consequences (e.g., reduced confidence and avoidance) may increase vulnerability to further WPV.

### 5.1. Theme 1—Forms and Manifestations of WPV Experienced by New Nurses

Reviewed studies identified WPV as a prevalent issue affecting new nurses, manifesting in various forms of aggression and inequities across systemic, interpersonal and professional domains. These experiences were categorised into five analytical subthemes: systemic hierarchical hostility and intimidation, social exclusion and isolation, verbal aggression and public humiliation, task overload and unfair workload, and sexual harassment and physical intimidation. Together, these subthemes describe WPV as both systemic (hierarchical hostility and workload inequity) and interpersonal (exclusion, humiliation and harassment), illustrating how structural power dynamics are enacted through day‐to‐day interactions with new nurses.

#### 5.1.1. Systemic Hierarchical Hostility and Intimidation

Several studies highlighted systemic hierarchical hostility and intimidation as entrenched behaviours within healthcare environments, where power imbalances enabled senior staff to dominate and control new nurses [23, 31–35, 37, 38, 40, 42–45]. Senior incivility and intimidation were widely reported, with nurses experiencing persistent scolding, dismissive attitudes and excessive scrutiny, often reinforced through public reprimands and intimidating surveillance [23, 28, 31, 34, 38, 42, 43]. WPV was often systemic and culturally normalised, with hierarchical structures reinforcing hostility and initiation practices that positioned new nurses at the lowest ranks, subjecting them to harsh treatment [35, 37, 38, 44]. Studies further reported that bullying behaviours, such as unjustified corrections and institutionalised dismissiveness, were common and framed as “rites of passage” that new nurses had to endure to earn their place in the profession [40, 42, 45]. Additionally, some studies described deliberate undermining, where senior staff withheld critical information, misled new nurses or interfered with their workflows [32–35, 38]. For example, one nurse described intimidation through surveillance: *“She stood at the door with her arms crossed, glaring us down… watching for mistakes… I felt nervous…”* [42]. This illustrates how surveillance and fear of error enacted hierarchical power and undermined early confidence.

#### 5.1.2. Social Exclusion and Isolation

Social exclusion and isolation were identified in the literature as mechanisms that marginalised new nurses within healthcare teams, preventing their full integration into the workplace [31–33, 35, 38–43, 45]. Team exclusion was a recurring theme, with studies documenting instances where new nurses were deliberately ignored or marginalised by colleagues and senior staff [31, 33, 42]. Neglect and lack of support further exacerbated isolation, as senior staff often refused to assist new nurses, expecting them to manage independently even in challenging clinical situations [32, 38, 41]. Several studies described silent hostility and indifference, where experienced staff withheld greetings, avoided giving positive feedback or maintained emotional detachment, creating an unwelcoming work environment [38, 40]. Social shaming and gossip also reinforced exclusion, with new nurses experiencing public ridicule, gossip about personal issues and discriminatory remarks, particularly related to linguistic or cultural differences [32, 43, 45]. Lastly, discouragement of input and questions was commonly reported, as new nurses faced dismissive reactions or ridicule when seeking guidance, often silencing them in discussions about patient care [31, 32, 34, 35, 38, 39]. For example, one nurse stated: “*You’re new… you’re on your own… You earn your salary just as I earn it, why do I have to do your job?*” [38]. This positioned help‐seeking as illegitimate and intensified isolation during transition.

#### 5.1.3. Verbal Aggression and Public Humiliation

Verbal aggression and public humiliation, as highlighted in eight reviewed studies, emerged as overt and interpersonal manifestations of WPV [31, 32, 34, 35, 39, 41–43]. Managers and senior staff frequently reprimanded or criticised new nurses in public settings, including in front of colleagues, doctors, patients or families, constituting overt forms of verbal aggression and public humiliation [31, 32, 39, 41, 42]. Sarcastic comments and name‐calling were also commonly reported, with senior staff using dismissive language [35, 43]. Studies additionally highlighted instances of verbal aggression by physicians, including shouting, expressing frustration or criticising nurses’ decisions [34, 41, 43]. For example, one nurse recalled being “*yelled at… directly in front of the patient… belittle[d]… The patient requested I not be her nurse… It was so embarrassing.”* [41]. This shows how humiliation extended beyond the nurse to affect care interactions and perceived credibility.

#### 5.1.4. Task Overload and Unfair Workload

Reviewed studies highlighted the inequitable delegation of tasks as a significant manifestation of WPV experienced by new nurses [32, 33, 35, 39]. The findings indicated that inequitable task delegation often placed an excessive burden on new nurses, as senior staff avoided routine duties, leaving them to manage complex patient care, injections, administrative work and rounds alone [33, 39]. This was further exacerbated by unfair patient assignments, where new nurses were disproportionately assigned labour‐intensive tasks or dependent patients, while senior staff handled less demanding responsibilities [39]. Some studies also highlighted that when errors or task failures occurred under these conditions, new nurses were often blamed [35, 39]. Additionally, unsafe staffing practices disproportionately affected new nurses, particularly those perceived as young, single, or without family responsibilities, leading them to be assigned additional shifts beyond their capacity [32]. For example, one nurse described: “The elderly professional nurse will just sit by the nursing station the whole day… you will do rounds… injections… books… admin duties… she doesn’t assist you.” [33]. This reflects how unfair delegation increased fatigue, blame risk and reduced learning opportunities.

#### 5.1.5. Sexual Harassment and Physical Intimidation

Sexual harassment and physical intimidation were documented as recurrent forms of WPV in reviewed studies [41, 44]. Sexual harassment involved persistent advances, vulgar behaviour and inappropriate physical contact from senior doctors and patients. Instances included inappropriate messages, physical gestures and unwelcome touching during patient care [44]. Physical intimidation was also documented, ranging from unprofessional touching in public‐facing roles to severe threats of harm, including verbal threats, social media harassment and attempted physical aggression by senior staff or colleagues [41, 44]. For example, one nurse stated: “*I got harassed from my senior doctor… He teased me… tries to touch me… He knew that I would not complain*.” [44]. This highlights how gendered power dynamics and fear of disclosure intensified vulnerability for newly graduated nurses.

### 5.2. Theme 2—Coping Strategies of New Nurses Facing WPV

Reviewed studies indicated that new nurses employed various coping strategies to navigate WPV, demonstrating resilience and adaptability in hostile work environments. These strategies were categorised into four analytical themes: seeking social and professional support, adopting emotion‐focused coping mechanisms, engaging in avoidance coping and self‐preservation, and utilising problem‐focused coping and self‐improvement strategies. Together, these analytical themes reflect a continuum from engagement‐based coping (support‐seeking and problem‐solving) to self‐protective coping (emotion‐focused regulation and avoidance), shaped by hierarchical power dynamics and perceived risk of retaliation in the workplace.

#### 5.2.1. Social and Professional Support

Multiple studies reported that new nurses sought social and professional support as a primary coping strategy against WPV [15, 22, 31, 37, 39, 42, 43]. Team‐based support was found to reduce stress, as nurses felt a greater sense of belonging and security when colleagues provided encouragement and debriefing after critical incidents [31, 37]. Beyond the workplace, personal and informal support from family, friends and peers played a vital role in emotional coping, offering reassurance and shared experiences that helped nurses process workplace challenges and maintain resilience [22, 39, 42, 43]. Additionally, some nurses sought professional counselling support, with therapy providing psychological relief and structured coping techniques to manage distress, particularly in severe cases where emotional strain became overwhelming [15]. For example, one nurse described the protective role of peer support during acute distress: “*I kind of had a breakdown right there and luckily my friend was on that shift and she helped me, but if I didn’t have her, I felt like I would have just drowned*.” [42]. This illustrates how interpersonal support served as a stabilising resource during overwhelming workplace stress.

#### 5.2.2. Emotion‐Focused Coping

Reviewed studies indicated that new nurses used emotion‐focused coping strategies to regulate distress associated with WPV, employing both adaptive and maladaptive approaches [15, 22, 39, 43]. Adaptive strategies included temporary emotional relief techniques through socialisation, leisure activities and distraction methods, which allowed nurses to momentarily detach from workplace hostility [22, 43]. Some nurses also engaged in reframing, attempting to rationalise or downplay workplace mistreatment to maintain motivation at work [39, 43]. However, studies also documented maladaptive coping strategies, including crying in isolation, emotional eating and alcohol consumption, as mechanisms to release or suppress distress in response to persistent hostility [15, 22, 39]. For example, one nurse described repeated emotional release in private spaces: “*I cried every single day I worked. I would go into the bathroom to cry… You just go into the bathroom and cry… and move on.”* [15]. This reflects how emotional coping became a routine mechanism for enduring ongoing hostility.

#### 5.2.3. Avoidance Coping and Self‐Preservation

The reviewed literature identified avoidance as a prevalent coping strategy among new nurses experiencing WPV, allowing them to evade conflict, criticism and retaliation in hierarchical healthcare environments [34, 38–41, 43–45]. Avoiding confrontation was a key approach, as nurses withheld responses to aggression, remained silent in temporary roles and avoided interactions with senior staff to minimise friction [34, 39, 41, 43]. Many nurses also suppressed questions and avoided seeking clarification to prevent judgement from colleagues or preceptors, while others endured mistreatment to protect job security [38, 40, 45]. Ultimately, avoidance often manifested as suffering in silence, with nurses counting down days until rotations ended while navigating WPV through withdrawal and disengagement [34, 39, 45]. For example, one nurse described being advised to remain silent to avoid worsening the situation: “*My preceptor told me, ‘Don’t bother saying anything because she’s [the bully] a pet… It’ll just make you look bad… Just suck it up and take it.’*” [45]. This illustrates how workplace hierarchies and perceived organisational inaction–reinforced silence and self‐preservation.

#### 5.2.4. Problem‐Focused Coping and Self‐Improvement

The findings from multiple studies suggested that problem‐focused coping and self‐improvement strategies were actively employed by new nurses to counteract WPV by enhancing their skills, resilience and workplace adaptation [22, 37–39, 43]. To cope with violence at work, nurses sought to develop skills and knowledge by independently studying at home, observing senior colleagues and mastering advanced care techniques, while also internalising responsibility to prevent errors and regrets by remaining accountable for patient safety and their clinical growth [37–39, 43]. Resilience and professional perseverance were also emphasised, as nurses set daily goals, remained determined despite adversity and framed workplace hardship as an opportunity for personal growth [39, 43]. Beyond individual efforts, structural advocacy and adaptation were evident, with some nurses modifying communication styles, reflecting on past experiences and advocating for organisational support [22, 38, 43]. For example, one nurse described self‐directed learning as a way to build confidence and demonstrate capability: “*I would quickly go home and search for it… I have to prove it to the other nurses that I’m confident… and I’m actually capable of doing this.”* [39]. This highlights how competence‐building functioned as an active coping response to scrutiny and workplace hostility.

### 5.3. Theme 3—Consequences of WPV on Nurses’ Personal and Professional Lives

Reviewed studies highlighted that WPV had significant consequences on the personal and professional lives of new nurses, affecting their well‐being, job performance and career trajectories. These consequences were categorised into five themes: emotional and psychological impact, erosion of confidence and professional identity, compromised patient care and professional standards, career regret and retention challenges and physical health and personal life disruptions. Collectively, these consequence subthemes capture cascading effects from internal distress (psychological and identity impacts) to external outcomes (patient care compromises and intentions to leave), showing how WPV extends from individual harm to professional practice and workforce sustainability.

#### 5.3.1. Emotional and Psychological Impact

Studies reported that WPV led to severe emotional and psychological distress among new nurses [15, 23, 31, 36, 37, 39, 40, 43, 45]. Stress and anxiety were frequently cited, as nurses struggled to navigate hostility from colleagues and senior staff [31, 45]. Some studies further documented burnout, depression and PTSD‐like symptoms resulting from prolonged exposure to workplace hostility [15, 39, 43]. In extreme cases, workplace mistreatment led to suicidal thoughts, illustrating the profound emotional toll of WPV [15, 39]. For example, one nurse described the cumulative psychological toll of daily hostility: “*When you constantly deal with little stuff like that every day, you start to get anxious about goin’ to work because you don’t know who gonna come at you that day.”* [45]. This highlights how ongoing WPV created anticipatory anxiety that permeated nurses’ daily lives.

#### 5.3.2. Erosion of Confidence and Professional Identity

Multiple studies identified that WPV significantly undermined nurses’ confidence and professional identity, creating long‐term self‐doubt and career dissatisfaction [33, 34, 36, 37, 39, 42, 45]. Several studies described how repeated criticism and mistreatment from senior staff resulted in self‐doubt and feelings of incompetence [34, 39, 42]. Nurses reported feeling pressured to present themselves as competent professionals despite being denied meaningful learning opportunities, leading to frustration and a diminished sense of professional identity [33, 42, 45]. In response, some withdrew from workplace interactions due to fear of judgement, while hierarchical structures and exclusion from peer networks further exacerbated the feelings of powerlessness and isolation [36, 37]. For example, one nurse described the immediate impact of bullying on self‐perception: *“I went home… in tears… she had just totally broken my confidence down and I felt like I was totally incompetent.”* [42]. This illustrates how WPV directly undermined emerging professional confidence during a critical developmental stage.

#### 5.3.3. Compromised Patient Care and Professional Standards

The reviewed literature highlighted that WPV negatively impacted nurses’ ability to maintain professional standards and deliver safe, high‐quality patient care [31, 35, 38–41, 45]. Many nurses reported that fear of criticism and excessive workloads led them to adopt task‐focused care, prioritising efficiency over thoroughness, which increased the risk of patient harm [35, 39]. Studies also documented how negative work environments and poor communication resulted in incomplete handovers, miscommunication and errors that further jeopardised patient safety [38, 40, 41]. Additionally, persistent anxiety and emotional exhaustion diminished nurses’ ability to focus on patient needs, reinforcing the detrimental impact of WPV on healthcare standards [39, 45]. For example, one nurse reflected on how WPV altered care delivery: “*I couldn’t do my best… I didn’t do anything extra… I became task oriented, and it affected my patient care.”* [39]. This demonstrates how workplace hostility translated into diminished care quality and professional fulfilment.

#### 5.3.4. Career Regret and Challenges to Retention

The findings from multiple studies indicated that WPV contributed to career dissatisfaction, regret and increased turnover for newly graduated nurses who experienced it [32, 33, 38–40, 42]. Experiences of bullying, exclusion and excessive scrutiny during the early stages of practice led many to question their career choice and express doubts about continuing in the profession [32, 42]. Some nurses described feelings of shame and regret, particularly when they struggled to meet unrealistic workplace expectations [38, 42]. The toxic nature of workplace environments contributed to early career attrition, with some nurses leaving their units or the profession entirely due to lack of managerial support and persistent mistreatment [32, 40]. Moreover, WPV discouraged new nurses from recommending the profession to others, further exacerbating workforce retention issues [39, 40]. For example, one nurse described the intensity of turnover consideration during orientation: “*During my 3 months of orientation, I was bullied… set up to fail purposely. I considered leaving almost daily.*” [32]. This highlights how early exposure to WPV undermined retention before nurses had fully transitioned into practice.

#### 5.3.5. Physical Health and Personal Life Disruptions

Reviewed studies highlighted the toll of WPV on the physical health and personal lives of new nurses [23, 38, 39, 42]. The findings indicated that exposure to workplace hostility was associated with physical symptoms such as chronic pain, sleep disturbances, weight loss and persistent anxiety [38, 39, 42]. Additionally, several studies reported that WPV disrupted work–life balance, with nurses experiencing emotional exhaustion that extended beyond the workplace, straining family relationships and personal responsibilities [23, 39]. Guilt over limited time for childcare and home life further compounded stress, highlighting the far‐reaching consequences of WPV beyond the professional setting [23]. For example, one nurse described the physical manifestations of ongoing distress: “*You get into that vortex… you start sweating, you start feeling sick, you don’t sleep at night.*” [38]. This illustrates how WPV produced embodied stress responses that affected nurses’ health and daily functioning beyond work.

### 5.4. Synthesis of Themes: A Conceptual Model of WPV Dynamics Experienced by New Nurses

**FIGURE 2:**
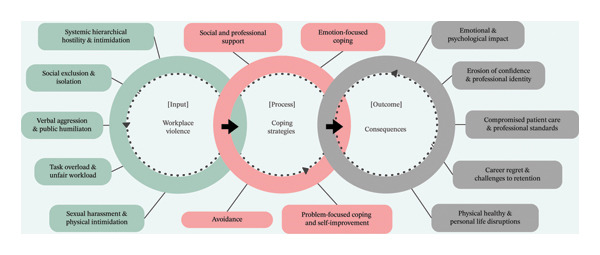
Conceptual model: workplace violence dynamics experienced by new nurses.

This meta‐synthesis introduces a conceptual model (Figure 2) to illustrate the cyclical and systemic nature of WPV experienced by new nurses. The model integrates the three meta‐themes identified in this review: forms/manifestations of WPV (Theme 1), coping strategies (Theme 2) and consequences (Theme 3). This directly addresses the review aim of synthesising how WPV is experienced, managed and how its impacts are experienced. Collectively, the findings indicate that WPV operates as a cyclical process, which is synthesised in the conceptual model below. The model conceptualises WPV as an ongoing cycle, where systemic inequities and interpersonal aggression (Input) trigger coping mechanisms (Process) that may provide temporary relief but fail to dismantle the underlying cultural and structural drivers of workplace hostility. Consequently, nurses experience enduring professional and personal consequences (Outcome), which, in turn, reinforce and sustain WPV.

The Input phase represents the systemic and interpersonal origins of WPV, including hierarchical hostility, social exclusion, task overload and other aggressive behaviours. These are not isolated incidents but rather patterns embedded within institutional hierarchies and cultural norms, making WPV a persistent aspect of nurses’ experiences. The Process phase captures the coping strategies new nurses adopt, illustrating a dual reality: While seeking social support and problem‐focused coping provides temporary protection, strategies such as avoidance and emotion‐focused coping may inadvertently reinforce systemic inequities by leaving the root causes unchallenged. Even problem‐focused coping, despite its benefits, often fails to generate real change when met with structural resistance. This dynamic interplay between coping mechanisms and workplace structures shapes how WPV is perpetuated over time. Finally, the Outcome phase demonstrates how WPV extends beyond the individual, affecting professional identity, patient care and workforce sustainability. Emotional strain, declining confidence and compromised professional standards create a self‐reinforcing cycle where the consequences of WPV leave nurses more vulnerable to further aggression while also hindering systemic reform.

By linking Input, Process and Outcome, the model demonstrates how the three meta‐themes are interrelated rather than independent categories, and how coping responses function within (and are constrained by) the broader system of WPV. Accordingly, addressing WPV requires more than individual resilience—it necessitates organisational and system‐level interventions that disrupt the cycle and target its structural drivers. Figure 2 visualises these dynamics, underscoring the need for interventions that disrupt the cycle at both the individual and organisational levels.

## 6. Discussion

This meta‐synthesis provides a comprehensive synthesis of qualitative evidence on WPV experienced by newly graduated nurses, offering deeper insights into its manifestations, coping strategies and consequences. The findings suggest that WPV is not merely an interpersonal issue but may be systemically embedded within healthcare settings, sustained by hierarchical structures, professional socialisation and an implicit culture of silence. Rather than being isolated incidents, WPV appears to be normalised in some institutions, shaping the ways in which new nurses experience and respond to aggression in the workplace. Importantly, this review contributes to what is already known by integrating qualitative findings into an interpretive pathway that links “what happens” (forms of WPV), “how new nurses respond” (coping) and “what follows” (consequences), rather than treating these as separate or parallel domains.

A key contribution of this synthesis is its conceptualisation of WPV as a cyclical and potentially self‐reinforcing process. Hierarchical hostility, exclusion and verbal aggression may not only serve as stressors but also function as informal initiation practices, where new nurses are expected to endure mistreatment as a “rite of passage” [46, 47]. This aligns with the theory of organisational silence by Morrison and Milliken [48], who suggest that institutions with weak reporting mechanisms or where incivility is modelled by senior staff could create an environment where workplace mistreatment is perpetuated rather than challenged. The findings of this synthesis add to the existing literature suggesting that toxic workplace cultures may contribute to staff burnout, decreased job satisfaction and increased turnover [49, 50]. However, beyond reaffirming these associations, our synthesis advances understanding by illustrating how these cultures are enacted in everyday practice through recurring mechanisms (e.g., surveillance, exclusion, public humiliation and workload inequity) that collectively position new nurses as vulnerable targets within hierarchical teams.

The paradox of coping strategies identified in this review further demonstrates how WPV may become entrenched in nursing practice. While some new nurses employ problem‐focused coping, such as developing clinical skills and seeking mentorship, others resort to avoidance, emotional detachment or passive endurance as survival strategies. These responses, though adaptive in the short term, may reinforce workplace hierarchies by discouraging open resistance or advocacy for systemic change [51, 52]. This reflects the concept of moral distress [53], where nurses are aware of injustices but feel unable to challenge them due to institutional barriers. The findings suggest that placing the onus on individual resilience alone may be insufficient, as it does not address the broader structural and cultural determinants of WPV. A distinct contribution of this review is highlighting that coping is not simply an individual choice but a constrained response shaped by perceived organisational inaction, fear of retaliation and dependence on senior staff for acceptance and learning opportunities. This helps explain why avoidance and silence persist even when nurses recognise WPV as unjust.

The consequences of WPV appear to extend beyond individual nurses to potentially impact patient safety, professional identity and workforce sustainability. Emotional exhaustion, self‐doubt and disengagement in new nurses may lead to hesitancy in clinical decision‐making, reduced communication and increased risk of errors, which could affect patient care [54–56]. Additionally, WPV has been associated with early career attrition, as some new nurses consider leaving the profession entirely due to persistent hostility and a perceived lack of organisational support [57, 58]. These findings reinforce the need to consider WPV not only as a workforce issue but also as a potential patient safety concern, requiring proactive organisational measures rather than solely individual‐level interventions. This review adds nuance by showing that consequences cluster and cascade, from psychological distress to reduced confidence, then to constrained communication and task‐focused care, while not implying a linear causal sequence. It helps clarify plausible pathways through which WPV may indirectly impact patient safety.

Current approaches to addressing WPV often focus on conflict resolution training, resilience‐building or peer support initiatives. While these interventions may be beneficial, they do not necessarily address the underlying structures that allow WPV to persist. This synthesis highlights the importance of broader institutional reforms, including stronger leadership accountability, effective antibullying policies, fostering psychological safety and ensuring accessible reporting mechanisms that are free from retaliation. Additionally, integrating WPV prevention strategies into nursing education and early career mentorship may equip new nurses with advocacy skills and organisational awareness, enabling them to navigate workplace challenges more effectively [13, 59]. A specific implication from this synthesis is that interventions should target both “inputs” (e.g., hierarchical hostility, exclusion and workload inequity) and the organisational constraints that shape coping (e.g., fear of retaliation and weak reporting cultures), rather than focussing only on strengthening individual resilience.

By synthesising diverse qualitative studies, this meta‐synthesis contributes to a broader understanding of WPV, shifting the discourse from individual experiences to systemic influences. Addressing WPV effectively may require a multilevel approach, where workplace culture, professional norms and institutional structures are proactively reshaped to promote respect, support and equity. Without targeted interventions that extend beyond resilience training, WPV may continue to undermine both nursing workforce retention and the overall quality of patient care. Overall, this review contributes by (1) integrating evidence into a coherent, cyclical model of WPV dynamics for new nurses and (2) clarifying intervention targets by identifying how workplace structures both generate WPV and constrain coping, thereby sustaining harm over time.

## 7. Limitation

This meta‐synthesis has several limitations. Although rigorous methodological processes were followed, the reliance on qualitative studies may limit the generalisability of the findings to broader populations. The included studies likely recruited participants who had experienced WPV, potentially amplifying negative experiences while underrepresenting those in more supportive environments. To mitigate this, the systematic and comprehensive search strategy aimed to capture diverse perspectives and contexts. The exclusive focus on English‐language, peer‐reviewed studies may have inadvertently excluded valuable findings published in other languages or formats. Additionally, as the synthesis relies on secondary data, the depth of analysis is inherently shaped by the quality and scope of the original studies. To address this, all included studies were critically appraised to ensure their scientific credibility and relevance. Variations in cultural and healthcare settings further complicate the applicability of the findings across different contexts. Finally, while the thematic synthesis provides a detailed framework for understanding WPV, the absence of quantitative prevalence data limits its ability to contextualise how widespread these experiences are among new nurses.

## 8. Conclusions

This meta‐synthesis provides a comprehensive synthesis of qualitative evidence on WPV experienced by newly graduated nurses, offering deeper insights into its manifestations, coping strategies and consequences. The findings suggest that WPV is not merely an interpersonal issue but may be systemically embedded within healthcare settings, sustained by hierarchical structures, professional socialisation and an implicit culture of silence. Rather than being isolated incidents, WPV appears to be normalised in some institutions, shaping the ways in which new nurses experience and respond to aggression in the workplace.

## 9. Relevance to Clinical Practice

Addressing WPV against new nurses is essential for improving retention, job satisfaction and patient care quality. The findings emphasise the need for systemic interventions that extend beyond individual coping strategies to institutional and policy‐level changes. Healthcare organisations must foster a culture of respect, accountability and psychological safety through structured mentorship, peer support programs and robust antibullying policies. Leadership engagement and training in conflict resolution can help create an environment where new nurses feel supported rather than marginalised. Additionally, integrating WPV prevention strategies into nursing education and professional development can better equip new nurses with the skills to navigate hostile environments. Shifting the focus from individual adaptation to systemic reform, organisations can enhance staff well‐being and overall patient care outcomes.

## Author Contributions

Gilny Rantung: conceptualisation, data curation, formal analysis, investigation methodology, and writing–original draft.

Yaping Zhong: conceptualisation, data curation, validation, investigation, methodology, and writing–review and editing.

## Funding

No funding was received for this manuscript.

Open access publishing facilitated by Monash University, as part of the Wiley ‐ Monash University agreement via the Council of Australasian University Librarians.

## Conflicts of Interest

The authors declare no conflicts of interest.

## Supporting Information

Additional supporting information can be found online in the Supporting Information section.

## Supporting information


**Supporting Information 1** Supporting File 1: ENTREQ Checklist. This file provides the completed Enhancing Transparency in Reporting the Synthesis of Qualitative Research (ENTREQ) checklist used to guide and report the qualitative synthesis process.

**Supporting Information 2** Supporting File 2: Example Search Strategy (MEDLINE). This file presents a sample of the search strategy, illustrating the specific MEDLINE database search terms and steps.

## Data Availability

The data that support the findings of this study are available from the corresponding author upon reasonable request.
