# Supplementary figures and images for: Experiences of Newly Graduate Nurses With Workplace Violence: A Qualitative Meta‐Synthesis
Source: J Nurs Manag. 2026 Mar 8;2026:4496213. doi: 10.1155/jonm/4496213 (PMC12968069; doi:10.1155/jonm/4496213)

Supplemental file 2: An example of the search strategy

**Medline**


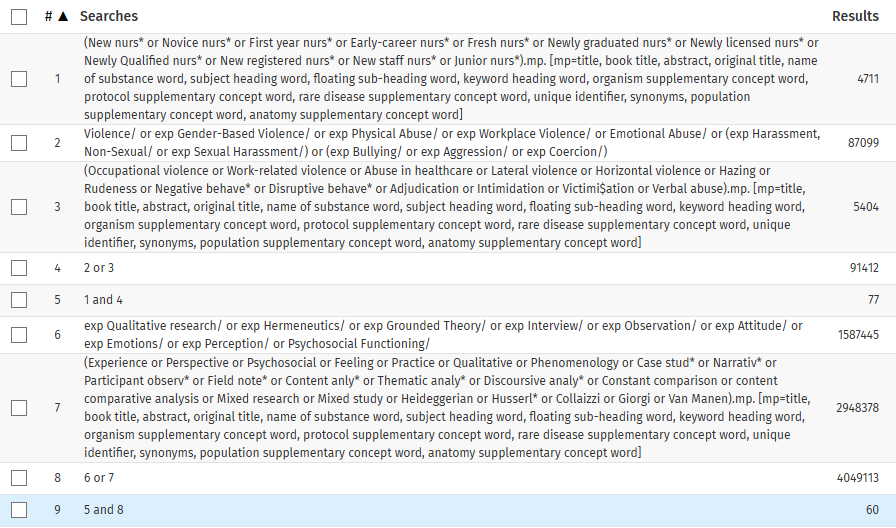

Supplement: Supplementary file 2 — Supporting Information 2 Supporting File 2: Example Search Strategy (MEDLINE). This file presents a sample of the search strategy, illustrating the specific MEDLINE database search terms and steps. [file JONM-2026-4496213-s001.docx]
